# An Autonomous Cannabinoid System in Islets of Langerhans

**DOI:** 10.3389/fendo.2021.699661

**Published:** 2021-07-05

**Authors:** Kanikkai Raja Aseer, Josephine M. Egan

**Affiliations:** Laboratory of Clinical Investigation/Diabetes Section, National Institute on Aging, National Institutes of Health, Baltimore, MD, United States

**Keywords:** endocannabinoids, cannabinoid receptors, β-cells, islet of Langerhans, obesity, diabetes

## Abstract

While endocannabinoids (ECs) and cannabis were primarily studied for their nervous system effects, it is now clear that ECs are also produced in the periphery where they regulate several physiological processes, including energy storage, glucose and lipid metabolism, insulin secretion and synthesis, and hepatocyte function. Within islet of Langerhans there is an autonomous EC system (ECS). Beta (β)-cells contain all the enzymes necessary for EC synthesis and degradation; ECs are generated in response to cellular depolarization; their paracrine influence on β-cells is mostly through the cannabinoid 1 receptor (CB_1_R) that is present on all β-cells; they modulate basal and glucose- and incretin-induced insulin secretion, and β-cell responses to various stressors. Furthermore, there is now accumulating evidence from preclinical studies that the autonomous islet ECS is a key player in obesity-induced inflammation in islets, and β-cell damage and apoptosis from many causes can be mitigated by CB_1_R blockers. We will thoroughly review the literature relevant to the effects of ECs and their receptors on β-cells and the other cell types within islets. Therapeutic potential of agents targeting EC/CB_1_R and CB_2_R is highly relevant because the receptors belong to the druggable G protein-coupled receptor superfamily. Present research in the ECS must be considered preliminary, especially with regards to human islet physiology, and further research is needed in order to translate basic cellular findings into clinical practice and the use of safe, clinically approved CBR modulators with and without glucose lowering combinations presently in therapeutic use for diabetes and obesity needs to be studied.

## Introduction

Healthy β-cells within the pancreatic islets of Langerhans, a storehouse of insulin, are poised to produce a rapid and sensitive response to acute changes in circulating blood glucose concentrations by first rapidly secreting insulin from the stored pool, and next quickly replenishing the pool by new insulin synthesis. However, impairment of the insulin response results from chronic overnutrition and unfavorable lifestyles, that, in turn, cause β-cell damage and diabetes. The physiology and mechanism of insulin secretion from β-cells is well orchestrated and involves a series of signaling events (1–3): 1) key glucose transporters, GLUT-2 in rodents and GLUT-1 in humans, allow the entry of glucose into β-cells; 2) intracellular ATP is then generated *via* glycolysis; 3) an increasing ATP/ADP ratio closes the inwardly rectifying ATP-sensitive potassium (K_ATP_) channels responsible for potassium ion efflux from the β-cells and for maintaining a resting membrane potential; 4) the resultant reduction in potassium ion efflux causes membrane depolarization and subsequent opening of L-type voltage-dependent calcium channels (VDCCs) allowing influx of calcium ions (Ca^2+^) into β-cells; 5) synchronous oscillations of elevated cytosolic Ca^2+^ and membrane excitability stimulate insulin release from β-cells into the systemic circulation. All of these events are modified by enhancing and inhibiting influences, which is where the ECS comes into play.

Much attention has been paid to the activation of membrane receptors of the G protein-coupled receptor superfamily in response to incretin secretion from the gut. This attention was warranted because it resulted in a new class of compounds, the glucagon-like peptide-1 receptor agonists (GLP-1R agonists), being now widely used to treat Type 2 diabetes (T2DM). They lower blood glucose because of increased insulin secretion and synthesis due to direct activation of adenylyl cyclase (AC) that results in increases in intracellular β-cell cAMP levels and downstream PKA activation. An added benefit is that their chronic use leads to a decrease in food intake because of a central action (4, 5). Furthermore, they have protective effects on the cardiovascular system (6–11). In contrast, there are inhibitory influences on insulin secretion due to activation of membrane G protein-coupled receptors, with somatostatin receptor activation being the most studied and clinically relevant (12, 13). More recently, it’s become clear that cannabinoid receptors (CBRs), which are also G protein-coupled receptors, are present in pancreatic islets and in β-cells specifically (14–23). We now also know that CB_1_R endogenous ligands and synthetic agonists contribute to inhibition of glucose-stimulated insulin secretion (GSIS) due to inhibition of AC, and they are involved in β-cell adaptation (and maladaptation) to prolonged nutrient overload and inflammation (18, 24, 25).

## General Background of ECs and CBRs

Endocannabinoids (ECs), anandamide (AEA) and 2-arachidonoyl glycerol (2-AG), are lipid messengers that are not pre-stored within cells but are synthesized and released on demand (26, 27). They are enzymatically produced by the essential enzymes, N-acyl phosphatidylethanolamine phospholipase D (NAPE-PLD) and diacylglycerol lipase (DAGLα), respectively, from membrane phospholipid-derived precursors containing arachidonic acid (a 20-carbon membrane phospholipid) or circulating fatty acid precursors and the rate of EC release depends on their synthesis rate. ECs are degraded primarily by fatty acid amide hydrolase (FAAH) and monoacylglycerol lipase (MAGL) and the degraded by-products are recycled back to cell membranes (27, 28). However, other enzymes implicated in the biosynthesis of AEA and 2-AG also exist, at least in mouse brain. Among these, 2-AG can be also produced by α,β-hydrolase-4 (ABHD4), the glycerophosphodiesterase-1 (GDE1), a soluble phospholipase A2, an unidentified phospholipase C, and phosphatases (29), whereas AEA can be produced by another isoform of DAGLβ (30). In addition, AEA can be degraded by one other enzyme, N-acylethanolamine acid amidase (31). EC synthesis rates are increased in both obese humans and rodents upon overconsumption of high caloric dietary meals rich in fats and sugars (19, 32). ECs have been found to be produced in adipose tissue, muscle (33, 34), and kidneys (35), all of which are involved in the control of energy metabolism. Moreover, ECs are also produced in gut, and they in turn at a minimum influence hepatic function and signaling through the vagal afferents (25, 36–39). Additionally, ECs are produced in stellate cells of liver (40) and islets of Langerhans: this review focuses on islets of Langerhans.

CBRs are G protein-coupled receptors, as mentioned above, that are present on cell and mitochondrial membranes (24, 41–47). CB_1_Rs were first described as being abundant in the pre-synaptic cells of the central nervous system (CNS) where they control retrograde synaptic signaling and regulate, amongst their many functions, appetite and reward response in the hypothalamus (44). CB_2_Rs are primarily considered to be associated with the immune system (42, 48). Apart from the classical CBRs, the non-classical receptors GPR55 and transient receptor potential vanilloid 1 (TRPV1) are present in high levels in the brain (49, 50) and relay signals intracellularly through Gα_13_ and G_q_ proteins (51). CBRs were subsequently also found to be present in mouse, rat, and human islets (14–21, 24, 52–56).

## EC Biosynthesis and Degradation in Pancreatic Islets, the Islet Cell Types Involved, and Islet Cell Localization of CBRs

Islets of Langerhans are clusters of endocrine cells embedded in the pancreas that control blood glucose homeostasis. An islet is comprised of insulin-secreting β-cells, glucagon-secreting alpha (α-) cells, somatostatin-secreting delta (δ-) cells, pancreatic polypeptide-secreting PP-cells (sometimes referred to as γ-cells) and sparce ghrelin-containing ϵ-cells. β-cells are the most predominant cellular component of islets in most species – ~80% in rodents and ~50–60% in humans-the other islet endocrine cell types therefore constitute a much smaller proportion of the islet mass (57). As regards PP- and ϵ-cells there are no data with reference to the ECS.

In 1986 it was first documented that delta-9-tetrahydrocannabinol (Δ^9^-THC), the primary psychoactive constituent of cannabis, was involved in modulating insulin secretion (58). In the early 1990s, CB_1_R and CB_2_R were successfully cloned (59), although an exhaustive search and comprehensive knowledge of their distribution throughout the body was not delineated at the time. More than 30 years have elapsed since the first discovery of Δ^9^-THC’s effects on insulin secretion. With the established involvement of ECS in peripheral metabolism it seemed reasonable that the ECS might have a role in islet function; this is now being explored by several research groups (16, 21, 24, 60–65). Studies have documented the existence of a functional ECS, including EC enzymatic machinery and CBRs, in pancreatic islets (as described below). But researchers involved in studying the ECS in islets have failed to reach a consensus on exactly which endocrine islet cell types other than β-cells synthesize ECs in a regulated manner. The presence of both CB_1_R and CB_2_R in islets of mouse, rat and human has also been documented, and again, exactly which cell types have which functioning receptors is not definitive (14–21).

Starowicz et al. demonstrated for the first time that the critical biosynthetic enzymes for EC synthesis (DAGLα and NAPE-PLD) were present in islets of mouse, and that they were mostly in α-cells, whereas degrading enzymes MAGL and FAAH seemed restricted to β-cells. The authors then suggested that AEA and 2-AG participate locally as autocrine and intra islet paracrine signaling molecules in the control of secretory products from islet cells (19). However, they did not report that AEA and 2-AG were actually synthesized or secreted from α-cells. Further studies on the occurrence and cellular distribution of biosynthesizing and metabolizing enzymes inside the islets of Langerhans of mouse, rat and human have now emerged. Concerning the synthesizing enzymes in mouse islets, DAGLα is expressed in fetal α-cells, however expression levels of the enzymes (DAGLα and NAPE-PLD) were reported to be higher in β-cells in mature islets compared with α-cells (19, 24, 55). Of the degrading enzymes, MAGL and FAAH, the former was reported to be present in both α- and β-cells (mouse fetal and adult), but the latter was restricted only to β-cells (19, 24, 55). There is a poor agreement between the mouse and rat islet degrading enzymes MAGL and FAAH defined by expression of the latter enzyme mostly in α-cells, with β-cells showing a weak labeling, and the former enzyme localized to δ-cells of rat islets (20). In reviewing the literature, no data was found on the expression of synthesizing enzymes in islets of rat. However, a high glucose ‘pulse’ elevated the levels of both AEA and 2-AG in RIN-m5F rat insulinoma cells, which could be relevant to the notion of EC synthesis in β-cells as a source of ECs in rat islets (32). The distribution of synthesizing enzymes in human islets seems similar to mouse islets (24) but expression levels in the different islet cell types is unclear. DAGLα and NAPE-PLD were reported to be more abundant in β-cells than in α-cells, with one of the metabolizing enzymes FAAH reported to be confined to β-cell-rich areas (16): another report however detected FAAH mostly in α-cells (20). DAGLα and MAGL are present in both β- and α-cells (24, 55, 66).

ECs are generated within β-cells in response to cellular depolarization. When isolated mouse and human islets were subjected to depolarization by either KCl or increasing glucose concentrations, increasing amounts of ECs both 2-AG and AEA, measured by tandem mass spectrometry analysis, were generated (24). This therefore clearly shows that β-cells are generating ECs in response to depolarization. Mouse fetal α-cells produce 2-AG and α-cells in human islets are also reported to produce both ECs (55) but the stimulus or control of regulated synthesis in α-cells, if any, unlike the case for β-cells, have not been reported. EC synthesis in δ-cells has not been reported. Of note, disruption of MAGL, the enzyme key to degrade 2-AG, caused substantial increases in insulin and glucagon release from isolated human islets, demonstrating an essential role of endogenous 2-AG signaling in mediating both β-cell and α-cell function (66). Together, these reports indicate that α- and β-cells are likely sources of EC (both AEA and 2-AG) production.

Both AEA and 2-AG were reported to be overproduced in the pancreas of diet-induced hyperglycemic obese mice as compared with normal diet-fed mice (32). After 3, 8, or 14 weeks feeding of a high fat diet (HFD) to mice, increased biosynthesis of ECs and their decreased degradation within β-cells occurred, which was reflected by increased AEA or 2-AG in the whole pancreas of mice (19, 32). In fact, the HFD led to increased expression of the biosynthesizing enzymes and decreased expression of the degrading enzyme, FAAH, in β-cells, in keeping with elevated pancreatic AEA or 2-AG levels (19).

With respect to CBRs, earlier reports, based on mouse studies, suggested that CB_1_R is expressed exclusively on α-cells (17–19). In addition, mouse islet α- and presumably non-β-cells, whose islet cell type was not exactly defined but had the morphology of α-cells, were found to express CB_2_R (14, 19, 67) while later studies [including those reported by Juan-Pico et al. (14)] suggested it was also expressed in β-cells (17, 19, 21), but less so in α-cells (17). However, using laser capture of specific islet cells, CB_1_R was found to be specifically enriched in adult mouse β-cells and subsequent studies confirmed these results (21, 24, 55). A few studies reported that δ-cells also contain CB_1_R (20, 67) and CB_2_R (67). In addition to the classical receptors, GPR55 (a non-classical cannabinoid receptor) was mainly reported to be expressed in β-cells and in a subset of α-cells but was absent or had extremely low expression in δ-cells (53, 54). Moreover, TRPV1 was reported to be present in fetal and mature β- and α-cells in mice (55).

In rat islets, there is also lack of clarity as to which cells express CBRs. Both α- and β-cells were reported to express both CBRs (15, 17, 68). In keeping with their mouse studies, Bermudez-Silva reported that CB_1_R is more highly expressed relative to CB_2_R expression in rat α-cells compared to β-cells (17). In contrast, a more recent study reported that the abundance of both CB_1_R and CB_2_R is much higher in β-cells than non-β-cells, whose cell types were not determined (64). There is evidence that CB_1_R is also present in δ-cells (20). In rat, despite disparities in the degree of receptor expression, all three islet cell types (α, β and δ) express CB_1_R but CB_2_R has been so far reported to be limited to α- and β-cells (15, 17, 64, 68). The non-classical receptor GPR55 has been reported to be specifically detected in β-cells (52) and TRPV1 tended to be localized to the core portion of rat islets (and therefore presumably β-cells) (56).

In human islets, CB_1_R was reported to be expressed in α-cells, but in only a small proportion of β-cells (16, 17). However, another group reported that CB_1_R appeared confined to δ-cells and was not expressed by either α- or β-cells (20). Other reports over the last decade have documented the presence of CB_1_R in both α- and β-cells of human islets (24, 55). CB_2_R was also enriched in δ- and β-cells but not α-cells (16, 17, 24) though this is far from definitive. Whereas β-cells and a majority of α-cells express the non-classical receptor GPR55, this receptor was absent or expressed less in δ-cells (54).

**Figure 1:**
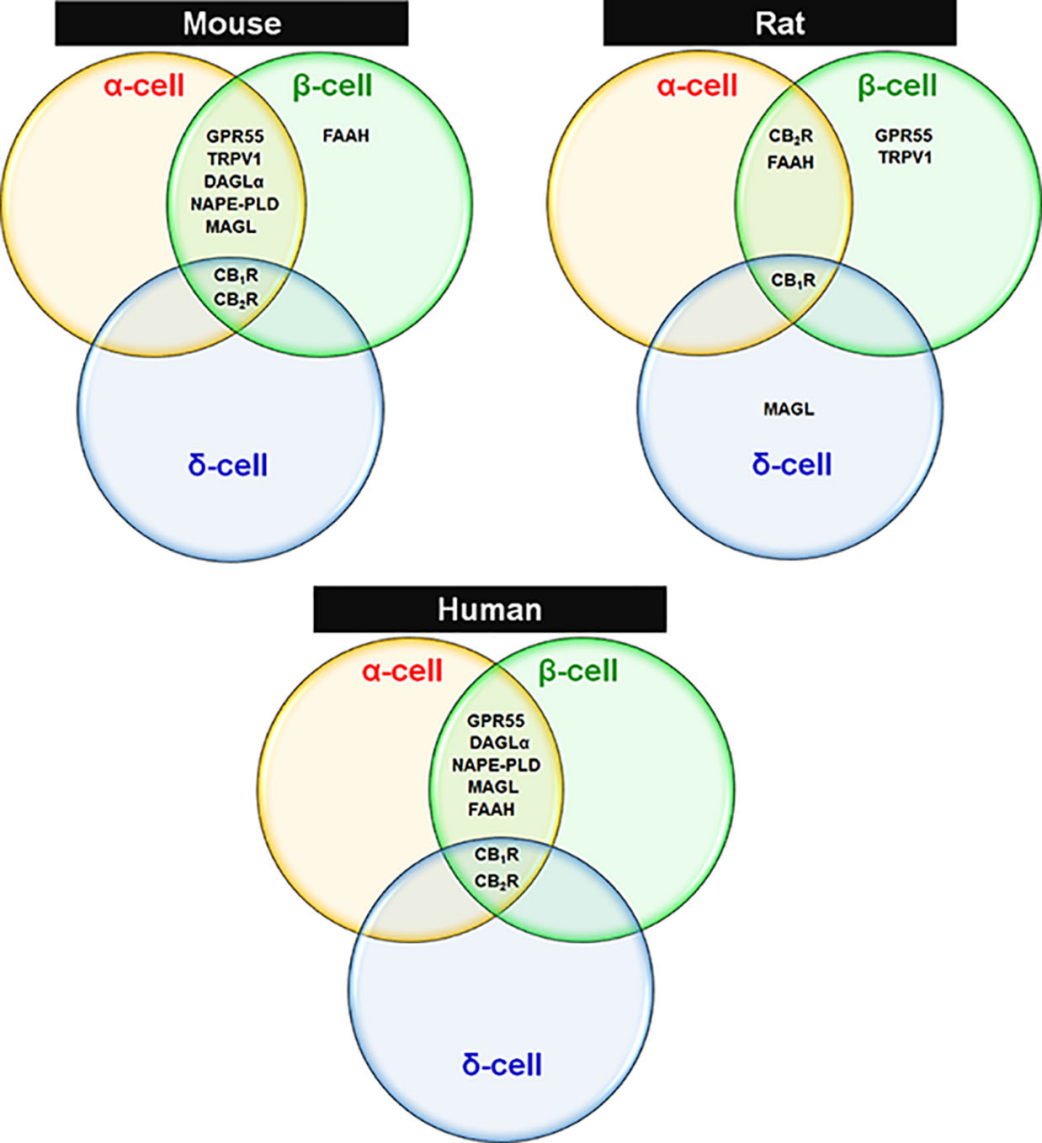
Expression of ECS elements in endocrine cells of mouse, rat and human islets. Venn diagrams are drawn based on reported findings and publications cited in **Tables 1** and **2** regarding species-specific and islet cell type-specific ECS components.

Overall, an extensive body of research has demonstrated that the EC components are present within the pancreatic islets of mice, rats and humans. Among these, the majority of enzymes implicated in the synthesis and degradation of ECs have indeed been detected mostly in α- and β-cells of mouse and human but exhibit a very limited spectrum in rat islets. However, demonstration of EC enzyme localization in δ-cells of mouse, rat and human islets is lacking. CBRs appear to be localized to both β-cells in the islet core and also in the islet mantel, where α- and δ-cells predominate across the three species. The existence and function of CBRs and EC enzymes have not yet been studied in PP- and ϵ-cells. The publications and reported findings concerning cellular expression of various components of the ECS in islet cells of mouse, rat and humans are summarized in **Tables 1**, **2**, and **Figure 1**. All this information is essential to have a complete picture of the importance of ECS in islets. Considering the apparent discrepancies between species, much exploration is required to further determine the detailed islet cell-type specific molecular profiling of ECS within pancreatic islets.

**Table 1:** Summary of similarities and differences of expression of biosynthetic and degrading enzymes in mouse, rat and human islets.

EC Enzymes	Islet cell types	Confirmed by	References
**Mouse**			
***Biosynthetic***			
**DAGLα**	α-cells	IF	(19)
	Mainly β-cells and less in α-cells	IF	(24)
	α-cells (fetal)	IF	(55)
**NAPE-PLD**	α-cells	IHC, IF	(19)
	Mainly β-cells and less in α-cells	IF	(24)
***Metabolizing***			
**MAGL**	β-cells	IHC, IF	(19)
	α- and β-cells	IF	(24)
	α-cells (fetal)	IF	(55)
**FAAH**	β-cells	IF	(19)
	β-cells	IF	(24)
**Rat**			
***Metabolizing***			
**MAGL**	δ-cells	IF	(20)
**FAAH**	Strong expression in α-cells and weak in β-cells	IF	(20)
**Human**			
***Biosynthetic***			
**DAGLα**	Mainly β-cells and less in α-cells	IF	(24)
	α-cells	IF	(55)
**NAPE-PLD**	Mainly β-cells and less in α-cells	IF	(24)
***Metabolizing***			
**MAGL**	Unreliable staining	---	(20)
	α- and β-cells	IF	(24)
	α- and β-cells	IHC	(66)
**FAAH**	Mainly α-cells and weak in β-cells	IF	(20)
	Mostly found in β-cell-rich areas	IF	(16)
	β-cells	IF	(24)

2-AG synthesis requires diacylglycerol lipase α (DAGLα), whereas AEA synthesis requires N-acyl phosphatidylethanolamine phospholipase D (NAPE-PLD). Degrading enzymes for 2-AG and AEA inactivation are MAGL and FAAH, respectively.

IF, immunofluorescence; IHC, immunohistochemistry.

**Table 2:** Summary of species-specific similarities and differences in CBR expression.

Expression of CBRs	Islet cell types	Confirmed by	References
**Mouse**			
	non-β-cells (undefined islet cell type)	ICC	(14)
	α-cells	IF, IHC	(19)
	α- and β-cells	IF	(18)
	δ-cells	IF	(20)
	α- and δ-cells	ICC	(67)
**CB_1_R**	Strongly stained in α-cells and to a lesser extent in β-cells	IF	(17)
	β-cells	IF, IHC	(21)
	α- and β-cells	IF, qRT-PCR (microdissected β-cells)	(24)
	β-cells (fetal)	IF	(55)
	β- and non-β-cells with α-cell morphology	ICC	(14)
	α- and β-cells	IF, IHC	(19)
	α- and δ-cells	ICC	(67)
**CB_2_R**	High in β-cells and to a lesser extent in α-cells	IF	(17)
	β-cells	IF, IHC	(21)
**GPR55**	β-cells	IF	(53)
	Confined to β-cells and in a small proportion of α-cells; Absent or low in δ-cells	IF	(54)
**TRPV1**	α- and β-cells (fetal and adult)	IF	(55)
**Rat**			
	β- and non-β-cells with α-cell morphology	IF	(15)
	δ-cells	IF	(20)
	Mostly in non-β-cells (undefined islet cell type) and in some β-cells	IF	(19)
**CB_1_R**	Strongly stained in α-cells and to a lesser extent in β-cells	IF	(17)
	α-cells	IF	(68)
	Increased abundance in β-cells and less so in non-β-cells (undefined islet cell type)	IF	(64)
**CB_2_R**	β- and non-β-cells with α-cell morphology	IF	(15)
	High in β-cells and low in α-cells	IF	(17)
	α-cells	IF	(68)
	Increased abundance in β-cells and less so in non-β-cells (undefined islet cell type)	IF	(64)
**GPR55**	β-cells	IF	(52)
**TRPV1**	Found localized to islet core (and therefore presumably β-cells)	IF	(56)
**Human**			
	Strongly stained in α-cells and to a lesser extent in β-cells	IF	(16)
	δ-cells	IF	(20)
**CB_1_R**	Strongly stained in α-cells and to a lesser extent in β-cells	IF	(17)
	α- and β-cells	IF	(24)
	β-cells	IF	(55)
**CB_2_R**	δ-cells	IF	(16)
	High in β-cells and low in α-cells	IF	(17)
	δ-cells	IF	(24)
**GPR55**	Confined to β-cells and most α-cells; Absent or low in δ-cells	IF	(54)

EC receptors consist of classical (CB_1_ and CB_2_) and non-classical (GPR55, TRPV1) receptors that have been identified in mouse, rat and human islets.

IF, immunofluorescence; IHC, immunohistochemistry; ICC, immunocytochemistry.

## Receptor Regulation and Function in β-Cells

A cardinal feature of CBR physiology is that constitutively synthesized CB_1_R is by no means a static component of the cellular machinery; rather approximately 70% of CB_1_Rs have an elimination half-life of approximately 5 hours while at least 30% of the receptors have a half-life as long as 24 hours, based on kinetic studies in neuroblastoma cells (69). CB_1_R expression inversely correlates with depolarization-induced increases in cytosolic Ca^2+^ influx, which is permissive of insulin secretion and synthesis, as mentioned above (24, 70, 71).

**Figure 2:**
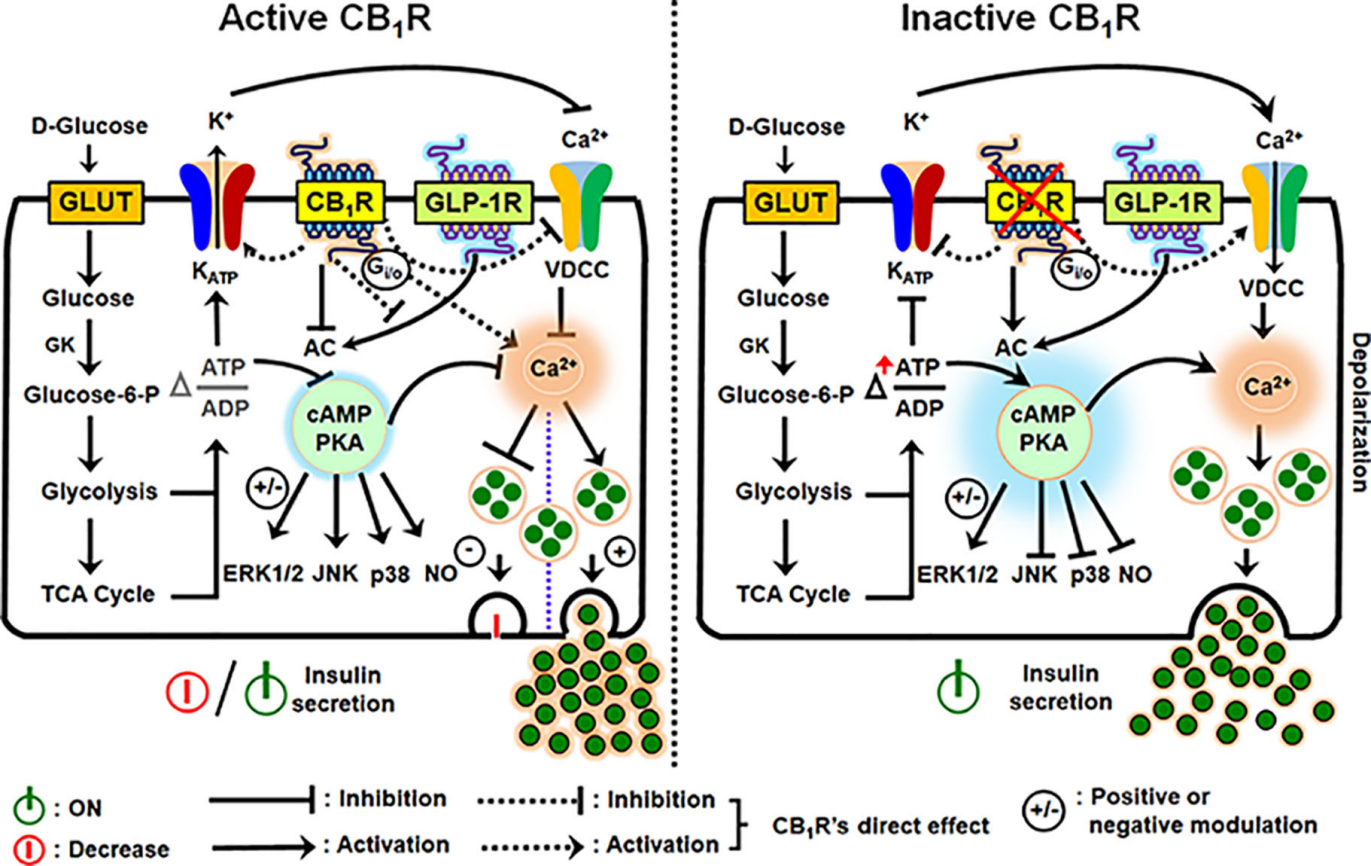
Schematic diagram illustrating the downstream events of CB_1_R activation and inhibition in the regulation of insulin secretion from β-cells. An increase in the intracellular glucose levels, which is transported by facilitated diffusion through glucose transporters, triggers the first permissive step in glucose-induced insulin secretion (GSIS). Glucose is phosphorylated by glucokinase (GK), the rate-limiting enzyme of glucose metabolism in β-cells, ATP is subsequently generated by glycolysis and the TCA cycle, causing an alteration in the intracellular ATP:ADP ratio. Increases in the [ATP/ADP] ratio closes the K_ATP_ channels and depolarizes the plasma membrane. The resultant opening of voltage-dependent calcium channels (VDCCs) and rise in cytosolic Ca^2+^ concentration allows for insulin exocytosis from β-cells. Although an increase in Ca^2+^ concentration is the primary or permissive insulin secretory event, cAMP production and protein kinase A (PKA) activity are critical in the fine tuning and potentiation of insulin secretion. The two gut-derived incretins, glucose-dependent insulinotropic peptide (GIP) and glucagon-like peptide -1 (GLP-1), are the main activators of adenylyl cyclase (AC) and subsequent cAMP formation; increases in cAMP augment GSIS by as much as 100% in non-diabetic subjects (GLP-1R is in the illustration: GIP has similar effects on AC). However, when CB_1_R is active, it signals through G_αi/o_ protein, inhibits AC, and attenuates cAMP production and protein kinase A (PKA) activity. CB_1_R also exerts influence on the K_ATP_ channel, decreasing potassium efflux, thereby inhibiting calcium entry through VDCCs because of hyperpolarization of the plasma membrane. In conjunction with the other forgoing events, downstream of AC-cAMP-PKA activity, CB_1_R activation impacts G-protein-dependent mitogen-activated protein kinases (MAPKs; ERK1/2, JNK and p38) and nitric oxide (NO) signaling, and the incretin receptors, GLP-1R and GIPR. The depicted receptor activation within the β-cell either inhibits or potentially stimulates insulin secretion under the influence of receptor-specific agonists *via* several mechanisms: the stimulatory role of CB_1_R in β-cell secretion, when reported, appears to be coupled to their capacity to increase intracellular calcium levels and reduction in cAMP production, whereas reduced intracellular cAMP formation can account for the inhibitory role of CB_1_R in insulin release.

Since ATP synthesis, K_ATP_ and VDCC are obvious targets at the proximal and distal rate-limiting or rate-potentiating steps of the insulin secretion under the control of calcium entry into β-cells (as described above), and with the on-going type 2 diabetes epidemic there is at present a keen interest in more fully elucidating all the molecular components that affect β-cell stimulus-secretion coupling, insulin synthesis and preserve β-cell function and mass. When CB_1_R is activated, it signals through G_αi/o_ protein, thereby inhibiting adenylyl cyclase (AC) and cAMP formation with consequent reduction in protein kinase A (PKA). This slows the movement of the insulin-laden secretory granules to the cell surface. Simultaneously, K_ATP_ channels are hyperpolarized, that is, do not close so easily, and opening of Ca^2+^ channels (VDCC) that normally allow cytosolic Ca^2+^ influx is negatively impacted, resulting in further suppression of hormone secretion from β-cells (25, 62, 71). In addition, G-protein-dependent mitogen-activated protein kinases (MAPKs; ERK1/2, JNK and p38) and nitric oxide (NO) generation are impacted (72–74). Other CB_1_R post-receptor interactions in islet β-cells has been defined, including (1) the interactions of its G_αi_ subunit with the insulin receptor to form a heterotrimeric complex that directly impairs insulin signaling by inhibiting autophosphorylation of insulin receptors and thereby diminishes the trophic effects of insulin on β-cells (60); (2) EC-mediated CB_1_R activation tonically counters the insulin secretagogue actions of the incretins – glucagon-like peptide (GLP)-1 and glucose-dependent insulinotropic polypeptide (GIP) (25); (3) CB_1_R activation also alters other receptor activity besides strictly CBRs, such as TRPV1 *via* CB_1_R-dependent AC-PKA axis and/or other unclear mechanisms that in turn influence intracellular Ca^2+^ levels (56, 75–77). Thus, CBR effects on β-cell function are an important component of the receptor’s role in regulating calcium-evoked hormone secretion and glucose homeostasis owing to alterations in the diversity of signaling pathways and post-receptor mechanisms (**Figure 2**). In the next sections we will throw a spotlight on the different CB_1_R actions in the endocrine cells of pancreas and the involvement of this receptor in hormone secretion, β-cell mass and inflammation in the pancreatic islets.

## CB_1_R Action on Islet Hormone Release and Synthesis

### Regulation of Glucose-Stimulated Insulin Secretion (GSIS)

Support for the evidence that CB_1_R is a negative regulator of insulin secretion has been demonstrated by combining small molecule studies and genetic tools in *in vivo* models, perfused and isolated islets, and β-cell lines: all show that activation of CB_1_R results in suppression of GSIS, whereas absence of CB_1_R enhances GSIS. CB_1_R antagonist treatment led to restoration of GSIS in Zucker diabetic fatty (ZDF) rats (61). Similarly, genetic and pharmacological blockade of CB_1_R indicated increased insulin secretion in mice (62, 63). Many CB_1_R antagonists (SR141716A, JD-5037, LH-21, AM251) also directly increased insulin release from mouse islets, clonal rat β-cells, mouse insulinoma βTC6 cells, and human islets (47, 53, 78) and in isolated islets from both prediabetic and diabetic mice, concomitant with improved islet function (79). However, there are discrepancies in results, going back to 2006. This will be further discussed below. We will first discuss studies which demonstrate increased insulin secretion, mostly as a result of CB_1_R antagonism.

The EC 2-AG blunts calcium oscillations in mouse cultured islets: calcium oscillations are permissive to pulsatile insulin release from β-cells (14). Similar observations have been obtained in isolated mouse pancreatic islets during their incubation with AEA and its non-hydrolysable analogue R(+) methanandamide, suggesting that CB_1_R activation promotes these effects (14). Additionally, this inhibitory effect in mouse islets have been repeated with AEA and the CB_1_R-selective agonist arachidonylcyclopropylamide because they both suppressed GSIS in conjunction with a decrease in Ca^2+^oscillations (18). The foregoing reports are congruent with the PKA/cAMP system-mediated desensitization for insulin release (80). Finally, CB_1_R directly modulates insulin secretion in human islets, removed from any influences from whole body. This is supported by the observation that the CB_1_R antagonists JD-5037 and rimonabant potentiated insulin release during perfusion of freshly isolated human islets when added to a stimulatory glucose concentration in the perfusate with more profound stimulatory effects exerted by JD-5037 than rimonabant at similar concentrations (47).

While most studies observed the inhibitory effects of CB_1_R activation on insulin secretion, there is data suggesting stimulatory action of CB_1_R on insulin secretion by direct addition of endocannabinoids AEA and 2-AG and their synthetic analogs to human and mouse islet preparations (16, 21) and rodent insulinoma cells (22, 81). GSIS is deemed to involve cytoskeletal and focal adhesion remodeling, which are key to insulin granule fusion with plasma membrane and exocytosis. CB_1_Rs are thought to play a role in focal adhesion kinase (FAK) phosphorylation because in INS-1E insulinoma cells FAK activation downstream of CB_1_Rs was found to make an essential contribution to cytoskeletal reorganization allowing for control of insulin release and potentially hypersecretion (81, 82). More precisely, the exception might be the ability of CB_1_R to activate FAK, which signals *via* Akt/PKB and ERK1/2, and the consequent FAK-mediated insulin secretion plays a role in CB_1_R-mediated signaling events in insulin stimulation as described for INS-1E cells (81) likely. This would also favor the physiological importance of this mechanistic link between enhanced circulating and local EC levels, and hyperinsulinemia as seen in type 2 diabetes (81). The role of CBRs in the regulation of insulin release was also reported from isolated human islets in both low and high glucose concentrations (16). This report would seem to indicate that human islets are particularly positively responsive (increased hormone secretion) to CB_1_R agonism with selective compounds (ACEA, for example) and ECs (AEA and 2-AG) while the effects of CB_2_R are inhibitory (16). CB_1_R-mediated cellular desynchronization indicative of opposite patterns of calcium oscillation coupled with insulin secretion and changes in gap junctions (83) associated with electrical β-cell coupling (84) could be possible pleotropic mechanisms underlying elevated insulin release. Support for potentiation of GSIS has been demonstrated in primary mouse islets and MIN6 cells during incubations with CB_1_R agonists 2-AG and ACEA, and, interestingly, with the CB_2_R agonist JWH015 – opposite to what was already published with reference to CB_2_R agonist in 2008 (16). Activation of both receptors elevated intracellular Ca^2+^ levels while inhibiting cAMP – these changes with CB_1_R agonist were prevented by a CB_1_R antagonist AM251 (21, 22). In these studies, using mouse islets and MIN6 cells, the investigators found that the insulin secretory profiles are responsive to 2-AG and ACEA in the presence of 2 and 20 mM glucose incubations reflecting the positive impact on insulin secretion primarily exerted by CB_1_R stimulation (21, 22). These results would reconcile that vicious cycles of overstimulation of CB_1_R leads to hyperstimulation of insulin secretion, hyperglycemia, lipid accumulation and consequent adipocyte hypertrophy observed in situations of obesity (21). Matias et al. (2006) had also reported enhanced insulin release due to incubation of rat insulinoma cells with HU-210, a CB_1_R agonist, when maintained on high glucose, and this effect was reversed by treatment with CB_1_R antagonist rimonabant, supporting the idea of a stimulatory role for ECs on insulin secretion (32).

From the above findings, it can be suggested that the contribution of CB_1_ and 2-AG to islet physiology and energy homeostasis is multifaceted, depending on the physiological or pathological conditions of the animal (16). Activation of CB_1_ is capable of stimulating insulin and glucagon secretion concomitant with lipogenesis in the liver and adipose tissue. As a consequence, glucose instead of being taken up by the muscle is converted to glycogen, and hence is stored instead of being oxidized (85–88). The observed rise in insulin release is also likely to facilitate the incorporation of glucose into adipocytes, thus favoring lipogenesis (16). Thus, it is possible that such events contribute to reduced energy expenditure and increased energy storage in the face of CB_1_R activation (16). Because of the involvement of CB_1_ in energy balance, Bermudez-Silva et al. (2008) has proposed that CB_1_ can be considered a new physiological thrifty system and that the persistent activation of this receptor could predispose individuals to obesity and type 2 diabetes upon Western diet consumption (16). Therefore, in view of the discrepancies with reports of both potentiation and suppression of insulin secretion following CB_1_ or CB_2_ receptor stimulation, several mechanisms may account for the stimulatory and inhibitory role of CB_1_R in β-cell secretion (**Figure 2**).

### Effects on Incretin-Mediated Insulin Secretion

Another important determinant of insulin secretion are the incretins – GIP and GLP-1. They are gut hormones secreted from enteroendocrine cells into the bloodstream that greatly enhance glucose-stimulated insulin secretion in response to a meal ingestion (89). Once released into the circulation, GIP and GLP-1 activate their specific incretin receptors (GIPR and GLP-1R, respectively) on β-cells and stimulate AC activity (90, 91). Subsequently, a rise in intracellular cAMP is required for incretin-mediated insulin secretion (92). Incretin-mediated insulin secretion is inhibited by CB_1_R activation, and, *in vivo*, in addition to the enhanced early phase glucose-stimulated insulin secretion, mice deficient in CB_1_R have increased incretin secretion compared with control littermates (25). This suggests that CB_1_R, as a component of entero-insular axis, inhibits fasting in order to restrict their capacity to stimulate insulin secretion to only the fed state. Apart from directly influencing GSIS from β-cells, blockade of CB_1_R improves GLP-1-mediated insulin secretion in mice (25). Likewise, in the mouse insulinoma βTC6 cells and human islets, GLP-1R-mediated insulin secretion by exendin (Ex)-4 (a peptide agonist of GLP-1R) treatment was inhibited in a dose-dependent manner by both synthetic (ACEA) and endogenous (2-AG and AEA) CB_1_R agonists, indicative of negative EC actions in incretin-mediated insulin secretion by activated CB_1_R. The underlying mechanism for this likely is suppression of Ex-4-evoked increases in AC activity and reduced intracellular cAMP. Conversely, pharmacological abrogation of CB_1_R with either a global (AM251) or peripherally restricted (JD-5037) CB_1_R antagonist was reported to rescue such effects by releasing the ACEA/cannabinoid blockade of Ex-4 action in both mouse insulinoma cells and human islets (25). Similar observations have been obtained in βTC6 cells and human islets but with incubations in the presence of another synthetic CB_1_R agonist WIN55,212-2. Indeed, Ex-4-stimulated cAMP accumulation and insulin secretion from βTC6 cells was abolished by WIN55,212-2 in a dose-dependent manner, whereas AM251 enhanced the insulinotropic effects of Ex-4 in human islets (63). Such findings are consistent with the concept that CB_1_R negatively impacts incretin-mediated insulin secretion in rodents, isolated islets and β-cell lines.

### Regulation of Glucagon and Somatostatin Secretion

In diet-induced obese mice after overnight fast, intra-peritoneal (IP) and intra-cerebroventricular administration of rimonabant caused increased glucagon secretion with concurrent increase in serum concentrations of glucose following glucose challenge (93). It is noteworthy that administration to diabetic mice of the CB_1_R antagonist BAR-1 led to a biphasic pattern of insulin and glucagon gene expression: their levels were reported to increase after 4 weeks of BAR-1 treatment but decrease after 8 weeks (79). In isolated human islets, CB_1_R agonists (2-AG and ACEA) had stimulatory effect on glucagon and somatostatin release at low glucose concentrations and ACEA-mediated glucagon release were prevented by AM251 (16). This has physiological relevance because somatostatin, as mentioned above, is an inhibitor of insulin secretion while glucagon is necessary for gluconeogenesis in order to prevent hypoglycemia (glucagon is also a stimulant to insulin secretion: all β-cells and hepatocytes express the Gs protein-coupled glucagon receptor). Separate studies reported that incubation of human islets with ACEA for approximately 2 days elevated glucagon content, thereby resulting in increased basal glucagon secretion. However, ACEA incubation for 5 days appeared to have had no effect on basal or arginine-stimulated glucagon release from human islets (94).

## Regulation of β-Cell Mass and Turnover by CB_1_R

Islet β-cell mass, in general, is controlled by the dominant need for insulin to regulate metabolism (95) and it is regulated on the plus side by proliferation and alterations in cell size, and on the negative side by apoptosis and immune-mediated destruction. Reductions in β-cell mass has been observed in T1 and T2DM, with a more profound decline in T1DM (96, 97). Multiple lines of evidence point to CB_1_R as being on the negative side of β-cell mass regulation. Exposure of isolated human and mouse islets to two different CB_1_R antagonists SR141716A and AM251 led to increased β-cell proliferation that had first been stimulated by cytokines (98) and β-cell-specific CB_1_R null mice have bigger islets because of increased numbers of β-cells as do global CB_1_R null mice (55, 62). These observations are also consistent with the larger islets present in AM251-injected mouse (24). AM251 treatment allowed for improved β-cell recovery and mitosis of remaining β-cells after streptozotocin (STZ), a known β-cell toxin, was given to mice (60). Taken together, these studies suggest that activated CB_1_R negatively contributes to β-cell mass and proliferation.

Insulin is a known positive regulator of β-cell mass through IRS2/AKT downstream signaling (99). As regards mechanisms underlying CB_1_R effects on mass, the anti-trophic effects of CB_1_R activation in mice were reported to involve inhibition of insulin receptor autophosphorylation by interaction of the G_αi_ subunit of CB_1_R directly with the insulin receptor, which occurs by binding of G_αi_ to the activation loop of its tyrosine kinase domain (60). This is in line with the report that genetic and pharmacological disruption of CB_1_R enhanced insulin receptor signaling *via* IRS2/AKT in β-cells which led to increased β-cell mass (24). Additionally, CB_1_Rs regulate the expression of the anti-apoptotic protein Bcl-2 and cell cycle regulator cyclin D2 in pancreatic β-cells. Treatment of β-cell lines with WIN55,212-2 led to a decrease in the expression of Bcl-2 and cyclin D2 that in turn induced cell cycle arrest in G0/G1 phase and caspase-3-dependent apoptosis. On the contrary, genetic deletion and pharmacological blockade of CB_1_Rs after injury in mice led to increased levels of Bcl-2 and cyclin D2 in pancreatic β-cells (100).

## Regulation of Inflammation in Islets by CB_1_R

There is a growing consensus that disruption of CB_1_R in preclinical animal models of obesity and T2DM modifies and preserves β-cell function and mass. Because ECs are synthesized during glucose stimulation, their synthesis is increased with HFDs, and high fat/sugar feeding leads to β-cell apoptosis (60), the concept of a role for β-cell-derived ECs in intra-islet inflammation was investigated. At this point, both autocrine and paracrine inflammatory actions have been attributed to CB_1_R activation. Consistent with an anti-inflammatory role of CB_1_R disruption, β-cell-specific deletion of CB_1_R eliminated the effects of diet-induced inflammation in β-cells *via* negative modulation of oxidative stress and NLRP3 inflammasome activation and led to preservation of β-cell function and survival in mice (62). Additionally, pharmacological blockade of CB_1_R by JD-5037 protected β-cells from inflammation and loss and allowed for normal plasma glucose levels in ZDF rats (61). To add to the complexity of action of ECs, islets have resident macrophages and both CBRs reside in those macrophages (61, 62, 101). CB_1_R-stimulated isolated human and rodent macrophages induced activation of NLRP3-ASC inflammasome with concomitant induction of the macrophage chemotactic protein (MCP)-1 and the release of cytokines such as IL-1β and IL-18 that resulted in apoptosis of β-cells (61). And depletion of CB_1_Rs in infiltrating macrophages implicated the macrophage-expressing CB_1_Rs as causing β-cell inflammation and cell death during obese conditions rather than cell-autonomous signaling of CB_1_R in β-cells as the cause. As proof, when the receptor was selectively inactivated in the macrophages of ZDF rats *via* IP delivery of glucan-encapsulated CB_1_R siRNA particles, inflammatory changes were reversed, and normoglycemia and GSIS were restored as opposed to those in rats receiving scrambled siRNA. The consequent protective actions appeared to be due to suppression of the nucleotide binding domain-like receptor protein 3-apoptosis associated speck-like protein containing caspase activation and recruitment domain (CARD) inflammasome activation and resistance to IL-1β overproduction (61). Interestingly there are intriguing results in this inflammatory space using cannabidiol. Δ^9^-THC protected mice from STZ-induced hyperglycemia and protected against the onset of hyperglycemia in non-obese diabetic mice (NOD; a mouse model akin to human autoimmune-mediated T1DM), indicative of protection of β-cell function and survival in the face of toxin- and autoimmune-mediated inflammation. Chronic administration of Δ^9^-THC appeared to prevent oxidative damage and reduce inflammation by decreasing the expression of IFN-γ, TNF-α and IL-12 in whole islets (102–104). However, it’s not evident by which mechanism or through which receptors Δ^9^-THC may be having its effects. It is possible that activation of CB_2_R and/or GPR55, or both, was the underlying protective mechanism in islets: this space is worth watching.

## Conclusions

A growing body of evidence attests that an overactive peripheral ECS predisposes to the risk of developing diabetes and insulin resistance. Numerous studies that employ different approaches, animal models and *in vitro* systems have shown that an activated ECS inhibits basal insulin secretion, GSIS and incretin action. However, there are some studies that argue for stimulatory action of ECs and synthetic CB_1_R agonists in insulin secretion, at least with supraphysiological levels, that possibly plays a role in hyperinsulinemia as seen in obesity. Additionally, CB_1_R is involved in control of β-cell proliferation and islet mass, β-cell apoptosis, and toxin, inflammatory and immune-mediated β-cell destruction. There is evidence that chronic CB_1_R blockade (i.e., either genetic or pharmacological) ameliorates diabetes and insulin resistance and prevents β-cell loss in animal models of diabetes and obesity. Additionally, it prevented apoptosis of β-cells in isolated human islets. Clinical studies are now required to translate animal findings into treatments.

## Author Contributions 

JME and KRA for conceptualization, writing, and editing. Both authors discussed the content, reviewed and approved the submitted version.

## Funding

The authors are supported by the Intramural Research Program of the National Institute on Aging (NIA/NIH).

## Conflict of Interest

The authors declare that the research was conducted in the absence of any commercial or financial relationships that could be construed as a potential conflict of interest.

## Abbreviations

Δ^9^-THC, delta-9-tetrahydrocannabinol; 2-AG, 2-arachidonoylglycerol; AC, adenylate cyclase; ACEA, arachidonoyl-2′-chloroethylamide; AEA, anandamide; AM251, N(piperidin-1-yl)-5-(4-iodophenyl)-1-)2,4-dichlorophenyl)-4-methyl-1H-pyrazole-3-carboxamide; CB_1_R, Cannabinoid receptor type 1; CB_2_R, Cannabinoid receptor type 2; DAGLα, diacylglycerol lipase alpha; EC, endocannabinoid; ECS, endocannabinoid system; FAAH, fatty acid amide hydrolase; GLP-1R, Glucagon-like peptide 1 receptor; GPR55, G protein-coupled receptor 55; K_ATP_ channel, ATP-sensitive potassium channel; KO, Knockout; MAGL, monoacylglycerol lipase; NAPE-PLD, N-acyl-phosphatidylethanolamine phospholipase D; PKA, protein kinase A; SR141716A (rimonabant), 5-(4-Chlorophenyl)-1-(2,4dichloro-phenyl)-4-methyl-N-(piperidin-1-yl)-1H-pyrazole-3 carboxamide; T1DM, type 1 diabetes mellitus; T2DM, type 2 diabetes mellitus; TRPV1, transient receptor potential vanilloid 1; VDCC, Voltage-dependent calcium channel.
